# Design of Phosphonated Imidazolium-Based Ionic Liquids Grafted on γ-Alumina: Potential Model for Hybrid Membranes

**DOI:** 10.3390/ijms17081212

**Published:** 2016-07-27

**Authors:** Marie-Alix Pizzoccaro, Martin Drobek, Eddy Petit, Gilles Guerrero, Peter Hesemann, Anne Julbe

**Affiliations:** 1 Institut Européen des Membranes, UMR-5635 CNRS-UM-ENSCM, Université de Montpellier (CC047), Place Eugène Bataillon, F-34095 Montpellier cedex 5, France; marie-alix.pizzoccaro@etu.umontpellier.fr (M.-A.P.); martin.drobek@umontpellier.fr (M.D.); eddy.petit@univ-montp2.fr (E.P.); 2 Institut Charles Gerhardt, UMR-5253 CNRS-UM-ENSCM, Université de Montpellier (CC1701), Place Eugène Bataillon, F-34095 Montpellier cedex 5, France; peter.hesemann@umontpellier.fr

**Keywords:** imidazolium-based ionic liquids, γ-alumina, phosphonate coupling agent, grafting, solid state NMR, hybrid membrane

## Abstract

Imidazolium bromide-based ionic liquids bearing phosphonyl groups on the cationic part were synthesized and grafted on γ-alumina (γ-Al_2_O_3_) powders. These powders were prepared as companion samples of conventional mesoporous γ-alumina membranes, in order to favor a possible transfer of the results to supported membrane materials, which could be used for CO_2_ separation applications. Effective grafting was demonstrated using energy dispersive X-ray spectrometry (EDX), N_2_ adsorption measurements, fourier transform infrared spectroscopy (FTIR), and special attention was paid to ^31^P and ^13^C solid state nuclear magnetic resonance spectroscopy (NMR).

## 1. Introduction

In competition with amines, ionic liquids are known to interact strongly and reversibly with CO_2_, making supported ionic liquid (IL) materials versatile solids for use in adsorptive or membrane CO_2_ separation applications [1]. The most common systems are composed of ILs either impregnated or confined in matrices, which can be porous or non-porous (i.e., polymer, ceramic or hybrid matrices). These materials can have applications, for example, in batteries [2,3,4], as electrolytes [5] or as CO_2_ separation systems [6,7]. Imidazolium-based ILs grafted onto the surface of porous supports are promising systems for a range of applications, including catalysis [8,9], chromatography [10,11,12] and gas separation [13,14]. These types of systems have been defined by Fehrmann et al. [15], as supported ionic liquids (SILs), which refer to either inert or catalytically-active covalently-bound monolayers of ILs. In these materials, the IL does not act like the bulk liquid anymore, but as a surface modifier. As reported by the authors, tailoring the chemical nature of the support, as well as its microstructure (pore size, size distribution, surface area, etc.), govern the grafting of the IL and its distribution on the support surface. Covalent linking of ILs on a ceramic oxide support appears as an attractive strategy to fine-tune solids with outstanding properties for CO_2_ adsorption and with improved long-term stability. Ionic liquids can be grafted on mesoporous silica-based supports, such as MCM-41 [16] or SBA-15 [17], and they also can be incorporated within a silica hybrid matrix [18]. These mixed ionic-mineral phases have been the most widely-investigated systems for applications such as heterogeneous catalysis [13,19,20], gas separation [13,14] or CO_2_ sorption [21].

γ-alumina (γ-Al_2_O_3_) is a commonly-used ceramic support, and its hydroxylated surface is attractive for anchoring or grafting active species for either gas separation or heterogeneous catalysis [22]. In addition, this material can be cast easily as a continuous membrane film and was thus selected in this work as a relevant support for grafting imidazolium-based ionic liquids. Controlling the chemical grafting of ILs in the pores of a porous material is much more challenging than their simple impregnation in a porous support, yielding the supported ionic liquid phase (SILP). Obviously, the choice of the functionalized imidazolium-based IL is a key parameter, but the characterization of the grafting reaction is a tricky task. The efficiency of the grafting step needs to be quantified, and the spatial proximity between the grafted sites needs to be determined.

Several functionalized imidazolium-based ILs have been reported in the literature with functional groups, such as trimethoxysilyl, thiol-, ether-, carboxylic acid-, amino- and hydroxyl-groups [23]. Each of these functional groups is adapted for grafting on a pre-functionalized support. Vangeli et al. [14] selected the trimethoxysilyl group to react with the hydroxyl groups of silica-based materials pre-treated with a piranha solution. The grafting reaction has been performed in two steps: (i) grafting of a silylated precursor and (ii) quaternization with 1-methylimidazole, yielding the imidazolium species. Despite the detection of carbon by elemental analysis and the measured decrease of support specific surface area after grafting, the demonstration of both the quaternization reaction and anchoring configuration were rather unclear.

The chemical modification of γ-alumina powders with organosilanes has been largely investigated in the literature [24,25]. As an alternative, grafting reactions could also be realized with phosphonate or phosphinate coupling functions. Randon et al. [26] have linked phosphoric acid and alkyl phosphonic acid to the surface of both titania and zirconia membranes in order to improve their performance for the ultrafiltration of bovine serum albumin (BSA) proteins. Caro et al. [27] modified a γ-Al_2_O_3_ membrane top-layer with alkyl/aryl phosphonic acids, thus resulting in an organophobic behavior. Guerrero et al. [28,29] studied the grafting of phenylphosphonic acid and its ester derivatives on both γ-Al_2_O_3_ and TiO_2_ powders. The surface bonding modes were investigated by both diffuse reflectance infrared spectroscopy (DRIFT) and ^31^P solid-state magic angle spinning nuclear magnetic resonance spectroscopy (MAS NMR). The same authors also patented a process for modifying an inorganic substrate with organophosphorus coupling agents, relevant for antibacterial applications. In this work, imidazolium-based ILs with phosphonyl functional groups were used for their intrinsic antimicrobial properties [30].

**Figure 1:**
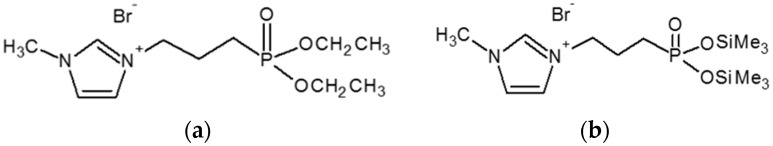
Representation of the structures of ImPE (1-methyl-3–(3–(diethylphosphinyl)propyl)-imidazolium bromide) (**a**) and ImTMSP (1-methyl-3–(3–((trimethoxysilyl)phosphinyl)propyl)-imidazolium bromide) (**b**).

The aim of the present work was to develop an optimized γ-Al_2_O_3_/imidazolium-based ILs system able to serve as a preliminary study for developing efficient gas separation hybrid membrane in which the IL will be effectively grafted on the pore surface. The approach involves the synthesis and characterizations of both the γ-Al_2_O_3_ support and the functionalized IL, followed by the investigation of the grafting reaction and the quantitative analysis of both the grafting step and the derived hybrid material. Two organophosphorus functionalized imidazolium-based ILs were selected (Figure 1): the 1-methyl-3–(3–(diethylphosphinyl)propyl)-imidazolium bromide (ImPE) and the 1-methyl-3–(3–((trimethoxysilyl)phosphinyl)propyl)-imidazolium bromide (ImTMSP). The synthesis of ImPE was carried out following the procedure described by Mu et al. [31], while the synthesis of ImTMSP was performed for the first time by adapting the work of McKenna et al. [32]. The modification of the γ-Al_2_O_3_ surface under either standard or forcing conditions was investigated by energy dispersive X-ray spectrometry (EDX) and N_2_ adsorption measurements. In addition, key information was derived from fourier transform infrared spectroscopy (FTIR) and ^31^P, ^13^C solid state NMR analysis. 

## 2. Results and Discussion

γ-alumina (γ-Al_2_O_3_) was prepared from boehmite using a sol-gel process described by Leenaars et al. [33] and followed by a 3-h thermal treatment in air at 600 °C. As revealed by ^1^H MAS NMR, hydroxyl groups are present on the alumina surface, and they are involved in the grafting reaction mechanism by condensation with ester functions (P-OX) of the organophosphonate coupling agent [34].

The surface of γ-Al_2_O_3_ powders was modified by treatment with an organic or aqueous grafting solution containing n-fold excess of either ImPE or ImTMSP ionic liquid. The quantity of IL used corresponds to the amount needed for a full surface coverage of the γ-Al_2_O_3_ particles (0.6 mmol, assuming an area of 25 Å^2^ per ionic liquid molecule). Depending on the ILs used, different reaction conditions were applied (Table 1). In order to evidence the spectroscopic characteristics of physisorbed phases or unreacted species on the surface of γ-Al_2_O_3_, a grafting experiment was first performed with ImPE in “physisorption conditions”. Secondly, in standard reaction conditions, grafting with ImPE was achieved during several days in an alcoholic solvent, while grafting with ImTMSP was realized during either one night or three days in dry methylene chloride. Otherwise, as reported for the grafting of diethyl phenylphosphonate coupling agents on γ-Al_2_O_3_, the use of forcing reaction conditions (i.e., excess of coupling agent relative to full surface coverage, and high temperature) did not lead to dissolution-precipitation mechanism (no formation of bulk aluminum phosphonate phases) and improved the surface grafting density [28,35]. Therefore, finally, forcing reaction conditions were also tested with ImPE in aqueous medium for one night by increasing the reaction temperature up to 130 °C. After the grafting treatment, samples were centrifuged, washed with an ethanol-water solution to remove unreacted and physisorbed species and dried at 70 °C under vacuum before analysis (see Materials and Methods). 

The characteristics of unmodified and grafted γ-Al_2_O_3_ powders are summarized in Table 2. Average phosphorus weight percentages (wt % P) obtained from EDX showed the presence of phosphorus in all of the grafted samples. The proportions do not exceed a full surface coverage (i.e., 3.2 wt % P), which is coherent with the surface reactions of the ILs coupling agents on γ-Al_2_O_3_. The sample modified with a six-fold excess of ImPE in forcing reaction condition (ImPE3) exhibited a slight increase of P contents by comparison with the sample obtained with a six-fold excess in standard condition (ImPE1). This result suggests that a higher temperature with a short reaction time tends to increase the rate of the surface modification reaction. For ImPE samples in forcing reaction conditions, an enhancement of ILs coupling agent excess in solution leads to an increase of the γ-Al_2_O_3_ surface coverage. In the case of ImTMSP samples, the P contents measured on modified γ-Al_2_O_3_ were in the range of the values obtained for the samples modified by ImPE. Nevertheless, no heat activation was needed with ImTMSP, suggesting a difference of reactivity, while the –PO(OSiMe_3_) function is known to be more reactive [28]. Finally, the efficiency of grafting reactions with ImTMSP did not seem to depend on reaction times, but was rather sensitive to the excess value of the IL coupling agent in solution. 

Nitrogen adsorption experiments did not reveal any important variation of the specific surface area values between the crude γ-Al_2_O_3_ powder and the grafted samples series, which is consistent with only the surface modification. The results of adsorption measurements gave also access to the BET constant (C_BET_) related to the affinity of the solid with N_2_ molecules ans so which is characteristic of the adsorbate/material surface interactions, as reported by Galarneau et al. [36]. The decrease of the C_BET_ value reflects a reduction of the enthalpy of adsorption of N_2_ molecules on the surface and thus gives qualitative information about surface modification. All of the grafted samples showed a lower C_BET_ constant than the starting γ-Al_2_O_3_ powders (Table 2). For each kind of coupling agent, the increase of the weight percentage of phosphorus measured on the modified samples correlates with a decrease of the C_BET_ constant. In addition, from the weight percentage of phosphorus and the specific surface area, we can estimate the grafting density (P nm^−2^) on the surface of the γ-Al_2_O_3_ powders, assuming an area of 25 Å^2^ per phosphonate molecule (Table 2). Therefore, a full surface coverage of the alumina particles should not exceed 4 P atoms by nm^2^. In all of the grafted samples, values from 0.6 to 1.4 of the grafting density were obtained, suggesting that the surface coverage does not exceed about 30% of the full monolayer in all cases.

In comparison with the literature, it has been demonstrated that the grafting on Degussa γ-Al_2_O_3_ in organic media with the diethyl phenylphosphonate coupling molecule resulted in a 50% surface coverage [28]. Moreover, in the same study, the authors have also evidenced that the grafting with bis(trimethylsilyl)ester phenylphosphonate coupling agent led to a higher percentage of phosphorus atoms, consistent with the formation of bulk aluminum phosphonate phases. It was also pointed out that the use of the dialkyl ester derivatives in organic media allowed the control of the grafting and excluded the formation of phosphonate phases even under prolonged heating. The partial surface coverage obtained in this study with the diethyl imidazolium phosphonate coupling molecule could result from a possible steric hindrance effect on the γ-Al_2_O_3_ surface (due to both the alkyl chain and the imidazolium ring) and also from the low reactivity of the coupling function. Furthermore, the results obtained with ImTMSP showed that the control of the reaction condition parameters can allow the incorporation of coupling agent quantities consistent with sole surface coverage without any evidence of the formation of bulk aluminum phosphonate phases. Thus, additional grafting parameters have to be tested (i.e., grafting duration, concentration, temperature, etc.) in order to optimize the reaction conditions and maximize the γ-alumina surface coverage.

**Figure 2:**
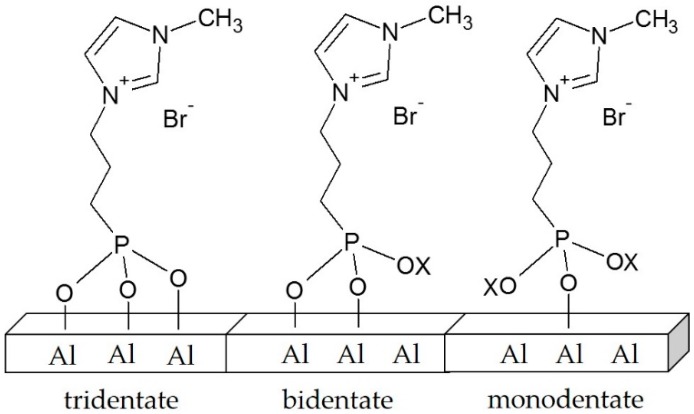
Schematic representation of some possible bonding modes for phosphonate coupling imidazolium-based ionic liquids on a γ-Al_2_O_3_ surface (X could be Et or SiMe_3_).

The reaction of organophosphorus derivatives on the γ-Al_2_O_3_ surface is supposed to involve both coordination of the oxygen of the phosphoryl groups (P=O) to Lewis acid sites and the condensation reactions of P-OX functions (X could be Et or SiMe_3_) with Al-OH surface groups. According to the literature, there are several possible bonding modes for phosphonate coupling molecules on an oxide surface [34]. In the case of phosphonyl imidazolium-based ILs, the possible bonding modes can be mono-, bi- or tri-dentate (Figure 2).

**Figure 3:**
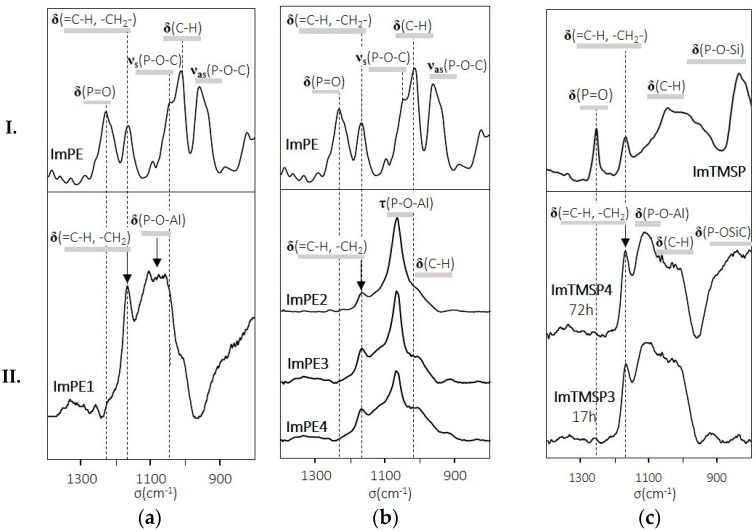
Experimental FTIR spectra of ImPE (I**a**,**b**) and ImTMSP (I**c**) and respective grafting on γ-Al_2_O_3_ prepared under different reaction conditions: (II**a**) with ImPE under standard condition (ImPE1), (II**b**) with ImPE under forcing conditions (130 °C, 17 h) at different concentration (ImPE2 to ImPE4: two- to 12-fold excess) and (II**c**) with ImTMSP under standard conditions (25 °C, six‑fold excess).

The FTIR spectra between 1400 and 800 cm^−1^ of the two organophosphonate functionalized imidazolium bromide-based ILs are presented in Figure 3I. ImPE and ImTMSP showed P=O stretching vibrations at 1230 cm^−1^ and 1251 cm^−1^, respectively, and C−H deformation vibrations at 956 cm^−1^ for ImPE and 1035 cm^−1^ for ImTMSP. Asymmetric and symmetric P-O-C stretching vibrations are present only for ImPE at 1042 and 958 cm^−1^ [28,37]. In addition, the spectra of ImTMSP present a P-O-Si deformation vibration at 833 cm^−1^. Starting from the ionic liquid structure, DFT calculation is useful to estimate the different vibration modes of the coupling agents (Figure S10 in the Supplementary Information) and to identify some of the deformation bands, such as the =C-H imidazolium band and the -CH_2_- spacer alkyl chain band present at about 1165 cm^−1^ for both ILs. In all grafted samples, we can notice the disappearance of the phosphoryl (P=O) stretching bands near 1230 and 1251 cm^−1^, suggesting that the phosphoryl oxygen is strongly bonded to Lewis acid surface sites by coordination (Figure 3IIa–c). Moreover, the IR spectra are dominated by an absorption band at 1171 cm^−1^, typical of the =C-H and –CH_2_– deformation bands of the imidazolium ring and the spacer alkyl chain. The PO regions of the grafted samples between 950 and 1250 cm^−1^ differ depending on both the IL and the applied reaction parameters. The IR spectra of samples treated in forcing reaction conditions with ImPE (Figure 3IIb) present a strong absorption band at 1065 cm^−1^, tentatively ascribed to the P-O-Al stretching vibration [37]. It can be noted that this band became gradually broader when increasing the quantity of grafted species. Therefore, the presence of weak absorption bands at about 1040 and 950 cm^−1^ (region of P-O-C stretching bands) does not preclude the existence of some P-OEt residual groups. 

The FTIR spectrum of the sample modified with ImPE under standard reaction conditions (Figure 3IIa) shows a broader absorption band around 1050 cm^−1^ corresponding to the P-O group stretching mode. This band could be attributed to P-O surface species in organophosphonate/metal oxide systems according to Quiñones et al. [38]. In addition, the presence of strong residual P-O-C stretching bands at about 1040 cm^−1^ cannot be excluded. The IR spectra of samples prepared with ImTMSP (Figure 3IIc) in standard conditions are quite similar to the ImPE1 spectrum. The most important difference comes from the presence of residual P-O-Si deformation vibration between ~1000 and 800 cm^−1^, which suggests that all of the coupling functions have not reacted with the alumina surface [28]. To conclude, IR spectroscopy of samples grafted in standard reaction conditions clearly reveals the presence of residual P-O-C or P-O-Si vibrations, stating that phosphonate units are preferentially linked to the alumina surface through bidentate (or monodentate) binding modes. In the case of samples prepared in forcing conditions, weak residual P-O-C stretching modes may be present on the infrared spectra, indicating that the dominating bonding mode of the phosphonate groups seems to involve tridentate PO_3_ units.

**Figure 4:**
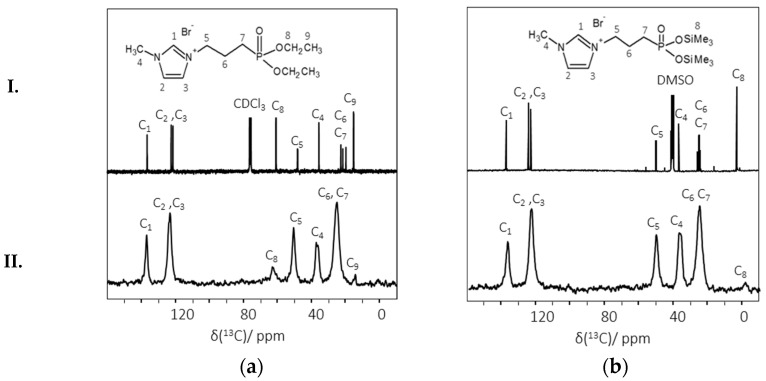
^13^C CP-MAS NMR spectra of pure ionic liquids (I) and the γ-Al_2_O_3_ grafted samples (II) prepared under the different reaction conditions. Comparison between: (**a**) pure ImPE and ImPE4 (130 °C, 17 h, 12-fold excess); (**b**) pure ImTMSP and ImTMSP4 (six-fold excess, 72 h).

^13^C cross polarization magic angle spinning (CP-MAS) liquid NMR spectra of ImPE and ImTMSP display both the specific chemical shifts (Figure 4Ia,b) of the 1-methyl-3-propylimidazolium group, numbered from C_1_ to C_7_, and those of the coupling functions, numbered from C_8_ to C_9_ for –POCH_2_CH_3_ and C_8_ only for –POSiMe_3_. In comparison, the ^13^C CP-MAS solid state NMR spectra of the grafted samples ImPE4 (Figure 4IIa) and ImTMSP4 (Figure 4IIb) show a slightly upfield shift for all of the atoms, principally due to the spatial proximity and chemical bonds with the γ-Al_2_O_3_ surface. Whatever the coupling function, the integrity of the organic molecule structure was conserved during the grafting process. In both grafted samples’ spectra, we can notice the presence of weak peaks attributed to residual -POCH_2_CH_3_ and -POSiMe_3_ functions, confirming the conclusions derived from the FTIR spectra (Figure 3) and supporting our hypothesis concerning the presence of multimodal bonding modes (i.e., tridentate, bidentate, monodentate). 

**Figure 5:**
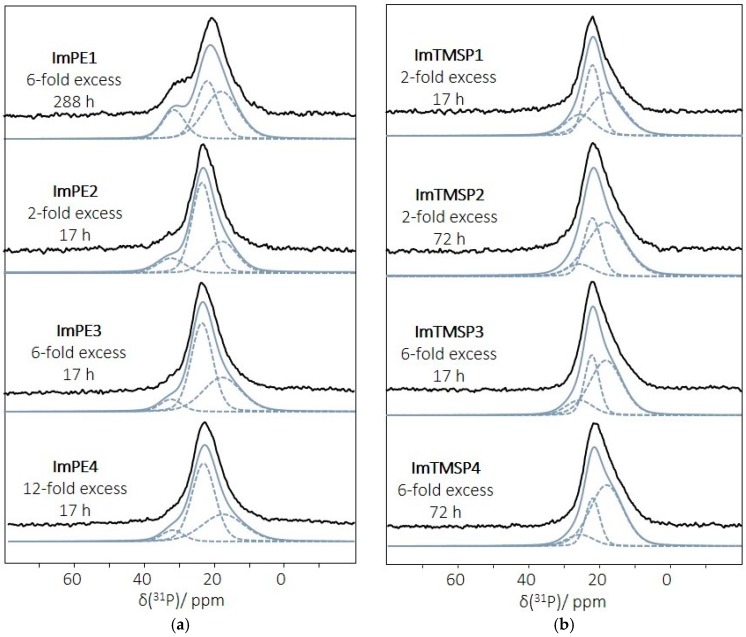
^31^P MAS NMR spectra of γ-Al_2_O_3_ grafted samples prepared under different reaction conditions: (**a**) standard conditions with ImPE (90 °C, 288 h) (ImPE1) and forcing conditions with ImPE (130 °C, 17 h) at different concentrations (ImPE2 to ImPE4: two- to 12-fold excess); (**b**) standard conditions with ImTMSP (25 °C) at various reaction times and concentrations: twofold excess, ImTMSP1 (17 h); ImTMSP2 (72 h); and six-fold excess, ImTMSP3 (17 h); ImTMSP4 (72 h).

Additional information was provided by the ^31^P MAS NMR spectra of the grafted γ-Al_2_O_3_. The powder treated under physisorption reaction conditions allows identification of the chemical shift corresponding to physisorbed species, with a sharp resonance at 32.1 ppm (Figure S11). The ^31^P MAS NMR spectra of all of the grafted samples (Figure 5a,b) did not reveal this peak, indicating that only grafted phosphonate species were present on the alumina surface. Moreover, we did not notice any additional upfield sharp resonance peak resulting from a dissolution/precipitation phenomenon and the formation of aluminum phosphonate bulk phases. This was confirmed by powder X-ray diffraction patterns (XRD) highlighting the amorphous structure of all of the grafted samples (Figure S12). 

The ^31^P MAS NMR spectra of the γ-Al_2_O_3_ powder modified with ImPE under forcing reaction conditions are displayed in Figure 5a (ImPE2, ImPE3 and ImPE4). All three spectra present a broad resonance centered at about 23.6 ppm. The simulation of the spectra indicated the presence of at least three sites (signals at 32.4, 23.6 and 17.9 ppm (Figure 5, Table 3)) revealing the presence of multiple bonding modes (Figure 2) for the phosphonate units as already discussed for the IR spectra. As reported by Brodard-Severac et al. [39], the interaction of the P=O groups with surface Lewis or Brønsted acidic sites should lead to a downfield shift. On this basis, the signal at 32.4 ppm, integrating from 7% to 13%, could be tentatively ascribed to the minor monodentate bonding mode (Figure 2). ^13^C CP MAS NMR indicated the presence of residual P-OEt functions, and IR spectra showed also weak residual P-O-C stretching modes, stating that the dominating bonding mode of the phosphonate groups seems to involve tridentate PO_3_ units. Therefore, we propose to ascribe the major signal at 23.6 ppm, integrating from 55% to 58%, to tridentate phosphonate PO_3_ units grafted on the γ-Al_2_O_3_ surface. The third resonance at 17.9 ppm, integrating from 29% to 38%, was then attributed to grafted phosphonate functions in a bidentate mode. For both ImPE3 and ImPE4, it is interesting to notice that the increase of the proportion of this bonding mode correlates well with the increasing intensity of the IR stretches of residual P-OEt functions. Consequently, by increasing the n-fold excess of coupling agents during the grafting reaction in forcing reaction conditions, both tridentate and bidentate bonding modes of the phosphonate units were favored.

The ^31^P MAS NMR spectrum of the γ-Al_2_O_3_ powder modified with ImPE under standard reaction conditions is also displayed in Figure 5a (ImPE1). It consists of a broad resonance centered at about 21.6 ppm with an important downfield asymmetrical shape. The simulation of the ImPE1 spectrum using a minimum number of resonances with a Gaussian–Lorentzian shape indicates the presence of at least three sites (Figure 5a, Table 3) at 31.6, 22.1 and 18.1 ppm, evidencing the presence of multiple bonding modes, as for ImPE2 to 4 (Figure 2). On the basis of the IR results, a higher number of residual P-OEt functions was detected when using standard rather than forcing reaction conditions. This implies an increasing proportion of monodentate and/or bidentate bonding modes of the phosphonate units. Thus, by comparison with the ImPE2 to 4 samples, we noticed a raising in the integration of the signals at 31.6 (18%) and 18.1 (45%) ppm, respectively ascribed to monodentate and bidentate bonding modes (Figure 2), which is in a good agreement with FTIR data. The signal corresponding to tridentate bonding mode at 22.1 ppm became minor with 37% integration. 

The above results suggest that the grafting of γ-Al_2_O_3_ with diethyl phosphonate coupling agent strongly depends on the surface modification reaction conditions. Soft standard reaction conditions mainly promote bidentate and monodentate bonding modes on the surface with a minority of tridentate phosphonate units. Conversely, forcing conditions lead rather to tridentate bonding modes on the alumina surface, with a smaller proportion of the other bonding modes.

Figure 5b displays the ^31^P MAS NMR spectra of γ-Al_2_O_3_ grafted under standard conditions (25 °C) using ImTMSP at different reaction times and quantities of IL (two- and six-fold excess). The spectra of ImTMSP1 to 4 samples are qualitatively similar and present a broader peak in comparison with the spectra of γ-alumina grafted with ImPE in forcing reaction conditions, centered at 22.1 ppm. In all cases, the NMR signals present an asymmetrical shape. According to the simulated spectra, three chemical shifts at 25.6, 22.1 and 18.1 ppm were identified with a major resonance for the latter (Table 4). As for the ImPE sample in standard condition with the presence of P-OEt groups, the IR spectra of ImTMSP1 to 4 revealed important residual P-OSiMe_3_ functions, implying an increasing proportion of monodentate and/or bidentate bonding modes of the phosphonate units. The liquid state ^31^P NMR spectrum of ImTMSP (Figure S8) indicated an upfield chemical shift at 24.7 ppm (to be compared with 29.8 ppm for pure ImPE (Figure S5)) in good agreement with the P-OEt to P-OSiMe_3_ conversion [29]. On this basis, the signal at 25.6 ppm, integrating from 9% to 18%, could be tentatively ascribed to the minor monodentate bonding mode with two P-OSiMe_3_ functions (Figure 2). As for the ImPE1 sample in standard reaction conditions, the major resonances attributed to phosphonate units in bidentate bonding mode correspond to the upfield chemical shifts located at 18.2 ppm and integrating from 47% to 67%. The last signal at 22.1 ppm could be ascribed to tridentate phosphonate units. By using ImTMSP as the coupling agent, we cannot correlate unambiguously the influence of the reaction parameters (excess of coupling agent, reaction duration) with the proportion of the different bonding modes.

The above results suggest that the reaction of γ-Al_2_O_3_ with either ImPE or ImTMSP in respective standard conditions promotes the grafting of phosphonate units with mainly a bidentate configuration. An increase of the temperature of the grafting reaction with ImPE favors the tridentate bonding mode of the phosphonate units on the γ-Al_2_O_3_.

## 3. Materials and Methods 

### 3.1. Starting Materials

Triethyl phosphite (98%), 1-methylimidazole (≥99%) and bromotrimethylsilane (BrSiMe_3_) (>97%) were purchased from Sigma-Aldrich (Saint-Quentin-Fallavier, France) and were used as received. 1,3-dibromopropane (98%) was provided by Fisher Chemical (Illkirch, France). Boehmite (Pural type) with a high crystallinity and surface area (249 m^2^/g) was supplied by CTI S.A. (Salindres, France). 

A batch of γ-Al_2_O_3_ powder was prepared by a sol-gel process based on colloid chemistry in aqueous media with a specific surface area of 220 m^2^·g^−1^. The γ-alumina powder batch was separated in small samples containing equal amounts of powder (400 mg) into an argon glovebox. This step avoided the presence of water on the alumina surface and yielded comparable conditions for the grafting reactions.

ImPE was obtained as a yellow oil in a high yield by the corresponding nucleophilic substitution of 1-methylimidazole with diethyl(3-bromopropyl)phosphonate in tetrahydrofuran (THF) (δ ^31^P = 29.80 (CDCl_3_)). 

ImTMSP was synthetized in a round-bottomed flask by the reaction of the ionic liquid ImPE with BrSiMe_3_ (3 equiv) in dry methylene chloride (CH_2_Cl_2_) (δ ^31^P = 24.70 (DMSO *d*_6_)). 

THF and CH_2_Cl_2_ were provided by Sigma‑Aldrich and dried with the PureSolv, Innovative Technology device.

### 3.2. Grafting Reactions

**Standard Reaction Conditions**: The “standard” reaction conditions are summarized in Table 1. Typical experiments are described below.

Grafting solution with ImPE was prepared by dissolving n-fold excess of the IL in the selected solvent. Five milliliters of the grafting solution and 400 mg of the γ-Al_2_O_3_ powder stored under argon were mixed in a glass bottle closed with a Teflon cap. The suspension was heated at 90 °C for 12 days. After cooling down to room temperature, the suspension was then centrifuged at 8500 rpm for 5 min using a Sigma 3-16P centrifuge equipped with a Sigma 12150-H rotor. The supernatant was removed, and the remaining powder was re-dispersed in 5 mL of a (1:1) ethanol-water solution to remove the physisorbed species from the surface, and the new suspension was stirred at room temperature for 5 min. The ethanol-water solution supernatant was removed after centrifugation (8500 rpm, 5 min), and this washing step was repeated twice. The resulting paste was then dried under vacuum (5 to 10 mbar) at 70 °C for ~16 h to afford the sample ImPE1 as a powder. 

Grafting with ImTMSP was performed directly in the grafting round-bottomed flask in dry CH_2_Cl_2_. Typically, ImTMSP (1.2 to 3.6 mmol, corresponding to a 2- or 6-fold excess relative to the amount necessary for a full surface coverage on the γ-Al_2_O_3_ particles) was dissolved in 15 mL of dry CH_2_Cl_2_ under stirring, and 400 mg of γ-Al_2_O_3_ powder stored under argon were dispersed in the grafting solution. The suspension was kept under stirring at 25 °C under argon for time periods ranging from 17 h to 3 days. The suspension was then centrifuged at 8500 rpm for 5 min and the supernatant removed. The remaining paste was re-dispersed in 5 mL of CH_2_Cl_2_, and the new suspension was stirred at room temperature for 5 min. After centrifugation, the CH_2_Cl_2_ supernatant was removed and the washing step repeated once. Then, the resulting pastes were washed with a (1:1) ethanol-water solution and dried in the same conditions as above, to afford the samples ImTMSP1 to 4 as powders. 

**Forcing Reaction Conditions**: The “forcing” reaction conditions are summarized in Table 1. Typical experiments are described below. 

Grafting ImPE solutions was prepared in water by dissolving the pure ImPE at different proportions (2-, 6- or 12-fold excess). Ten milliliters of the grafting solution and 400 mg of the γ-Al_2_O_3_ powder stored under argon were dispersed in an autoclave, which was closed with a Teflon cap. The autoclave was sealed and the suspension heated at 130 °C for 17 h. The resulting grafted powders were washed and dried as previously described for ImPE samples grafted under standard reaction conditions to afford the samples ImPE2 to 4 as powders.

### 3.3. Characterization

The BET specific surface areas and the C_BET_ constants of the samples were obtained from nitrogen adsorption experiments at 77 K by using a Tristar instrument (Micromeritics) for the grafted powders and a ASAP 2020 (Micromeritics) for the γ-Al_2_O_3_ powders. Prior to measurements, samples were degassed under vacuum overnight at 100 °C for the grafted powders and 300 °C for the γ-Al_2_O_3_ powder. The weight percentage of phosphorus in the samples was determined by EDX using a Zeiss scanning electron microscope (SEM) EVO HD15 at 10 kV equipped for EDX analysis with the AZtecEnergy analysis software (Oxford instruments. Abindong, UK). Samples were prepared as pellets for the analysis and deposited on double-sided carbon tape. FTIR spectra were obtained with a Perkin-Elmer Spectrum 2 spectrophotometer and were recorded in the 4000 to 400 cm^−1^ range using 32 scans at a nominal resolution of 4 cm^−1^ in ATR mode (spectrum of γ-Al_2_O_3_ as a background spectrum).

**Solution NMR experiments**: ^13^C and ^31^P NMR spectra were recorded using a Bruker 300-MHz NMR spectrometer at frequencies of 150.86 and 242.94 MHz, respectively. 

**Solid state NMR experiments**: Solid state NMR experiments were performed using a Varian VNMRS 600 MHz (14.1 T) NMR spectrometer. A 3.2-mm Varian T3 HX MAS probe was used for ^1^H, ^13^C and ^31^P experiments. The operating frequencies for ^1^H, ^13^C and ^31^P were 599.95, 150.86 and 242.86 MHz, respectively. All NMR experiments were performed under temperature regulation in order to ensure that the temperature inside the rotor is 20 °C.

^13^C CP-MAS solid state NMR spectra were recorded at a spinning frequency of 12 kHz MAS. Concerning the CP-MAS experiments, a contact time of 1 ms was fixed, the acquisition time to 30 ms, and the ^1^H channel was decoupling on this period. A recycle delay of 2 s was used with a number of scans of 11,450, which permit to obtain a signal-to-noise ratio between 30 and 35. ^13^C chemical shifts were referenced to external adamantane at 38.5 ppm.

^31^P MAS solid state NMR spectra were recorded at a spinning frequency of 20 kHz. The single pulse experiments were performed with a ~90° solid pulse of 3 μs and ^1^H decoupling during acquisition. A recycle delay of 45 s was employed (corresponding in both cases to full relaxation of ^31^P) with a number of scans of 56 for obtaining a signal-to-noise ratio between 53 and 79. ^31^P chemical shifts were referenced to external hydroxyapatite at 2.80 ppm (used as a secondary reference).

## 4. Conclusions

One important outcome of this study bears on the possibility to perform the grafting with imidazolium bromide-based ILs bearing phosphonate functions (ImPE or ImTMSP) on γ-Al_2_O_3_ powders, either in dry methylene chloride solvent (for ImTMSP) or in aqueous and alcoholic solvents (for ImPE). Compared to previous studies published in the literature describing the grafting of phenylphosphonic acid or its bis(trimethylsilyl)ester derivative, no bulk aluminum phosphonate phases were evidenced in the present work. Moreover, this study confirmed that the use of the diethyl imidazolium phosphonate coupling molecule allowed the control of the grafting reaction by using either prolonged heating or high temperature. Surprisingly, the same behavior was demonstrated with the bis(trimethylsilyl)imidazolium ester derivative.

FTIR and solid state NMR spectroscopy (^31^P, ^13^C) demonstrated that γ-Al_2_O_3_ surface modification with diethyl phosphonate coupling agent strongly depends on the grafting conditions.

Soft standard reaction conditions mainly promote bidentate and monodentate bonding modes on the surface, with a minority of tridentate phosphonate units. Conversely, the forcing reaction conditions mainly lead to the formation of tridentate bonding modes on the alumina surface, with a smaller proportion of the other ones. In addition, the grafting of ImTMSP in standard conditions seems also to promote the alumina surface modification by phosphonate units in a mostly bidentate configuration. 

However, in all cases, the surface coverage does not exceed about 30% of the full monolayer. The results obtained in this study for ImPE and ImTMSP could result from a possible sterically-hindered effect on the γ-Al_2_O_3_ surface (due to both the alkyl chain and the imidazolium ring) and to the low reactivity of the coupling function. Furthermore, the results obtained with ImTMSP suggest that additional grafting parameters have still to be tested (i.e., grafting duration, concentration, temperature, etc.) in order to optimize the reaction conditions and maximize the γ-Al_2_O_3_ surface coverage.

This work allowed establishing the optimized synthesis and characterization protocols for the development of imidazolium phosphonate-grafted γ-Al_2_O_3_ hybrid materials with controlled bonding modes and grafting rates. As a perspective on this fundamental work, the preparation and testing of the corresponding gas separation hybrid membranes will now be investigated. 

## Figures and Tables

**Table 1:** Standard and forcing conditions for grafting the γ-alumina powders with organophosphonate imidazolium-based ionic liquids (ILs).

	IL	Solvent (mL)	T (°C)	Time (h)	N-Fold Excess	N (ILs) mmol	Sample
Standard conditions	ImPE	2-butanol (5)	90	288	6	3.6	ImPE1
	ImTMSP	CH_2_Cl_2_ (14)	25	17	2	1.2	ImTMSP1
	ImTMSP	CH_2_Cl_2_ (14)	25	72	2	1.2	ImTMSP2
	ImTMSP	CH_2_Cl_2_ (14)	25	17	6	3.6	ImTMSP3
	ImTMSP	CH_2_Cl_2_ (14)	25	72	6	3.6	ImTMSP4
Forcing conditions	ImPE	water (10)	130	17	2	1.2	ImPE2
	ImPE	water (10)	130	17	6	3.6	ImPE3
	ImPE	water (10)	130	17	12	7.2	ImPE4

**Table 2:** Characteristics of the γ-alumina powders before and after grafting reactions.

Sample	C_BET_	wt% P ^a^	P nm^−2^ ^b^
γ-Al_2_O_3_	82	0	/
ImPE1	60	0.90 ± 0.05	0.9
ImPE2	63	0.62 ± 0.02	0.6
ImPE3	59	1.12 ± 0.04	1.1
ImPE4	55	1.42 ± 0.03	1.4
ImTMSP1	70	0.92 ± 0.04	0.9
ImTMSP2	66	0.92 ± 0.02	0.9
ImTMSP3	64	1.16 ± 0.10	1.1
ImTMSP4	63	1.00 ± 0.10	1.0

^a^ From EDX analysis; ^b^ average number of coupling molecules per nm^2^.

**Table 3:** Parameters used for the simulation of ^31^P MAS NMR spectra of γ-Al_2_O_3_ grafted with ImPE under either standard (ImPE1) or forcing (ImPE2, ImPE3 and ImPE4) reaction conditions.

Sample	ImPE1			ImPE2			ImPE3			ImPE4		
δ (ppm)	31.6	22.1	18.1	32.4	23.6	17.9	32.4	23.6	17.9	32.4	23.6	17.9
Width (ppm)	7.5	7.6	11.5	9.1	6.9	10.0	7.4	7.4	13.0	7.4	8.0	14.4
Integration (%)	18	37	45	13	58	29	7	55	38	7	57	36

**Table 4:** Parameters used for the simulation of ^31^P MAS NMR spectra of γ-Al_2_O_3_ grafted with ImTMSP.

Sample	ImTMSP1			ImTMSP2			ImTMSP3			ImTMSP4		
δ (ppm)	25.6	22.1	18.2	25.6	22.1	18.2	25.6	22.1	18.2	25.6	22.1	18.2
Width (ppm)	9.6	5.5	12	9	5.8	12.6	9	4.9	11.2	9	4.9	10.8
Integration (%)	18	35	47	9	30	62	10	23	67	13	29	58

## Supplementary Materials

The synthesis procedures and characterizations of γ-Al_2_O_3_ powder, the bulk 1-methyl-3–(3–(diethylphosphinyl)propyl)-imidazolium (ImPE), 1-methyl-3–(3–((trimethoxysilyl)phosphinyl)propyl)-imidazolium bromide (ImTMSP), as well as the details on XRD analysis, the synthesis procedure of the physisorbed sample, FTIR and DFT spectra of the ionic liquids, including DFT detailed calculations. The following are available online at Supplementary materials can be found at http://www.mdpi.com/1422-0067/17/8/1212/s1.
