# Supplementary material for: Design of Phosphonated Imidazolium-Based Ionic Liquids Grafted on γ-Alumina: Potential Model for Hybrid Membranes
Source: Int J Mol Sci. 2016 Jul 27;17(8):1212. doi: 10.3390/ijms17081212 (PMC5000610; doi:10.3390/ijms17081212)
Supplement: Supplementary file 1 [file ijms-17-01212-s001.pdf]

Supplementary Materials: Design of Phosphonated Imidazolium-Based Ionic Liquids Grafted on γ-Alumina: Potential Model for Hybrid Membranes

Marie-Alix Pizzoccaro, Martin Drobek, Eddy Petit, Gilles Guerrero, Peter Hesemann and Anne Julbe

Summary

1. Characterizations S2
2. Synthesis and characterizations of the γ-alumina powder S2
3. Synthesis and characterizations of phosphonyl imidazolium-based ionic liquids S3
4. ^{1}H, ^{13}C and ^{31}P NMR Spectra of the ionic liquids ImPE and ImTMSP S4
5. FTIR spectra of the ionic liquids in ATR mode and DFT calculated spectra S8
6. Physisorbed sample S8
7. XRD of γ-alumina and grafted powders S9

1. Characterizations

X-ray diffraction powder patterns were recorded using a PANanalytical X'Pert PRO diffractometer at the wavelength of Cu Kα (λ = 1.5405 Å) (X-ray power: 40 kV, 20 mA) in Bragg-Brentano scanning mode. The program scanned angles (2θ) from 4° to 50° with a 0.017° step, and a step time of 40 s.

Solution NMR experiments: ^{1}H, ^{13}C and ^{31}P nuclear magnetic resonance NMR spectra were recorded using a Bruker 300 MHz NMR spectrometer at frequencies of 300.13, 75.42 and 121.42 MHz, respectively. ^{29}Si NMR spectra was performed using a Bruker 400 MHz NMR spectrometer at frequencies of 79.46 MHz.

Solid state NMR experiments: Solid state NMR spectra were acquired on a Varian VNMRS 600 spectrometer (^{1}H: 599.95 MHz, ^{31}P: 242.86 MHz, ^{27}Al: 156.33 MHz). A 3.2 mm Varian T3 HXY magic angle spinning (MAS) probe was used for ^{1}H and ^{27}Al experiments, and a 3.2 mm Varian T3 HX magic angle spinning (MAS) probe was used for ^{1}H and ^{31}P experiments All NMR experiments were performed under temperature regulation in order to ensure that the temperature inside the rotor is 20 °C.

For ^{1}H experiments, the spinning frequency was 24 kHz, and the single pulse experiments were performed with a ~90° solid pulse of 2.5 μs. A recycle delay of 5 s was used (corresponding in both cases to full relaxation of ^{1}H). ^{1}H chemical shifts were referenced to external Adamantane at 1.80 ppm (used as a solid reference).

^{27}Al MAS NMR spectra at 14.1 T were acquired at a spinning frequency of 24 kHz. The single pulse experiments were performed with a ~15° solid pulse of 1 μs and ^{1}H decoupling during acquisition. A recycle delay of 5 s was used (corresponding in both cases to full relaxation of ^{27}Al). ^{27}Al chemical shifts were referenced to external $Al(NO_3)_3$ at 0 ppm.

^{31}P MAS solid state NMR spectra were recorded at spinning frequency of 20 kHz. The single pulse experiments were performed with a ~90° solid pulse of 3 μs and ^{1}H decoupling during acquisition. A recycle delay of 45 s was used (corresponding in both cases to full relaxation of ^{31}P) with a number of scan of 56 which permit to obtain a signal-to-noise ratio between 53 and 79. ^{31}P chemical shifts were referenced to external Hydroxyapatite at 2.80 ppm (used as a secondary reference).

2. Synthesis and Characterizations of the γ-Alumina Powder

The γ-alumina powder was prepared from the boehmite by a sol-gel process based on colloid chemistry in aqueous media according to the method reported elsewhere [33]. The boehmite (5 wt %) was placed in ultrapure water containing nitric acid (HNO_3/boehmite molar ratio ~0.042) as the peptizing agent. The suspension was sonicated during 15 min and then vigorously stirred for 2 days at room temperature. The resulting sol was centrifuged at 8500 rpm during 30 min in order to remove any unhydrolyzed particles. The as-obtained stable sol has been first concentrated by removing the water at temperatures under 150 °C, followed by thermal treatment at 600 °C/3 h in air, leading to the formation of γ-alumina powder (specific surface area of S_{BET} = 220 m²/g).

^{1}H solid state NMR measurements (600 MHz, 24 kHz): δ (ppm) −0.2 (μ^1-Al_{IV}); 0.5–2.8 (μ^2-Al_{VI}); 2.5–4 (μ^2-Al_{IV}, μ^3-Al_n); ^{27}Al solid state NMR (600 MHz, 24 kHz): δ (ppm) 10.2 (Al_{VI}); 68.2 (Al_{IV}). Attributions were assigned from the work of Taoufik et al. [40].

It must be noted that before each NMR measurement the powder has been re-calcined at 450 °C/3 h under nitrogen for removing any adsorbed water on the powder surface. All manipulation with the as-treated powder has been conducted in glovebox under an argon atmosphere.

3. Synthesis and Characterizations of Phosphonyl Imidazolium-Based Ionic Liquids

The diethyl(3-bromopropyl)phosphonate was synthesized via Arbuzov reaction from triethylphosphite and 1,3-dibromopropane, as described in [41]; its physicochemical constants coincided with the literature data. ^{1}H NMR (300 MHz, $CDCl_3$): δ (ppm) = 4.05 (m, 4H, –PO(C*H*$_2$CH$_3$)$_2$); 3.41 (t, 2H, Br–C*H*$_2$–CH$_2$); 2.07 (m, 2H, –C*H*$_2$–CH$_2$–P); 1.80 (m, 2H, –CH$_2$–C*H*$_2$–P); 1.27 (t, 6H, –PO(CH$_2$C*H*$_3$)$_2$). ^{31}P NMR (300 MHz, $CDCl_3$): δ (ppm) = 30.8.

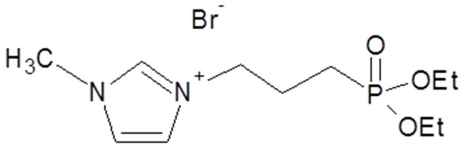

Figure S1. 1-methyl-3–(3–(diethylphosphinyl)propyl)-imidazolium bromide.

The 1-methyl-3–(3–(diethylphosphinyl)propyl)-imidazolium bromide (**ImPE**) was obtained as a yellow oil in high yield from the corresponding nucleophilic substitution of 1-methylimidazole with diethyl(3-bromopropyl)phosphonate adapted from the procedure published by Mu et al. [31]. The diethyl(3-bromopropyl)phosphonate precursor (27.00 g, 107 mmol) was dissolved in 50 mL of dry tetrahydrofuran (THF). Then, 1-methylimidazole (8.80 g, 107 mmol) was added dropwise and the mixture was heated to reflux at 70 °C during 3 days under argon. After cooling to room temperature and decanting, two phases could be distinguished as a yellow oil phase and a liquid phase. The two phases was separated and the yellow oil was washed twice with 50 mL of THF, then separated by liquid-liquid extraction with CH_2Cl_2 and H_2O. The combined organic fractions were solubilized in absolute ethanol, dried with anhydrous $MgSO_4$ and concentrated under vacuum to afford **ImPE** as a yellow oil with a 88% yield (32 g).

IR (ATR, cm^{-1}) ν: 3384, 3143, 3072, 2978, 1573, 1447, 1392, 1237, 1171, 1026, 965, 789, 475, 403.

^{1}H NMR (300 MHz, DMSO): δ (ppm) 10.53 (s, 1H, N–C*H*–N); 7.54 (s, 1H, N–C*H*); 7.36 (s, 1H, N–C*H*); 4.57 (t, 2H, C*H*$_2$–N); 4.06 (m, 4H, O–C*H*$_2$–CH$_3$); 4.06 (s, 3H,C*H*$_3$–N); 2.17-1.77 (m, 4H, C*H*$_2$–C*H*$_2$–P); 1.34 (t, 6H,O–CH$_2$–C*H*$_3$).

^{13}C NMR (300 MHz, $CDCl_3$): δ (ppm) 137.5; 123.7; 122.5; 62.0; 49.3; 36.7; 23.9; 22.8; 20.9; 16.5.

^{31}P NMR (300 MHz, $CDCl_3$): δ (ppm) 29.8.

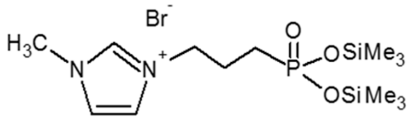

Figure S2. 1-methyl-3–(3–((trimethoxysilyl)phosphinyl)propyl)-imidazolium bromide.

The 1-methyl-3–(3–((trimethoxysilyl)phosphinyl)propyl)-imidazolium bromide (**ImTMSP**) was prepared in a round-bottomed flask by the reaction of the ionic liquid **ImPE** with $BrSiMe_3$ (3.5 equiv) in dry CH_2Cl_2. The following procedure is based on the procedure published by McKenna et al. [32]: 520 mg (1.52 mmol) of 1-methyl-3–(3–(diethylphosphinyl)propyl)-imidazolium bromide (**ImPE**) was dissolved in 10 mL of dry CH_2Cl_2. Then, $BrSiMe_3$ (0.82 g, 5.35 mmol) was added and the mixture was stirred at 25°C during 17 h under argon. At the end of the reaction time, the reaction mixture was concentrated under vacuum, leading to the expected IL as a one brown-orange paste in quasi quantitative yield. (98%, 633 mg).

IR (ATR, cm^{-1}) ν: 3153, 3064, 2956, 2894, 1563, 1448, 1251, 1164, 1045, 998, 835, 754, 728, 694, 646, 619, 505, 428.

^{1}H NMR (300 MHz, DMSO): δ (ppm) 9.08 (s, 1H, N–C*H*–N); 7.73 (s, 1H, N–C*H*); 7.66 (s, 1H, N–C*H*); 4.13 (t, 2H, CH_2–N); 3.79 (s, 3H,CH_3–N); 1.94-1.41 (m, 4H, CH_2–CH_2–P); 0.00 (s, 6H,O–Si–CH_3).

^{13}C NMR (300 MHz, DMSO): δ (ppm) 137.2; 124.2; 122.7; 49.6; 36.2; 25.5; 24.4; 23.7; 2.4.

^{31}P NMR (300 MHz, DMSO): δ (ppm) 24.7.

^{29}Si NMR (400 MHz, DMSO): δ (ppm) 7.8.

4. ^{1}H, ^{13}C and ^{31}P NMR Spectra of the Ionic Liquids ImPE and ImTMSP

ImPE

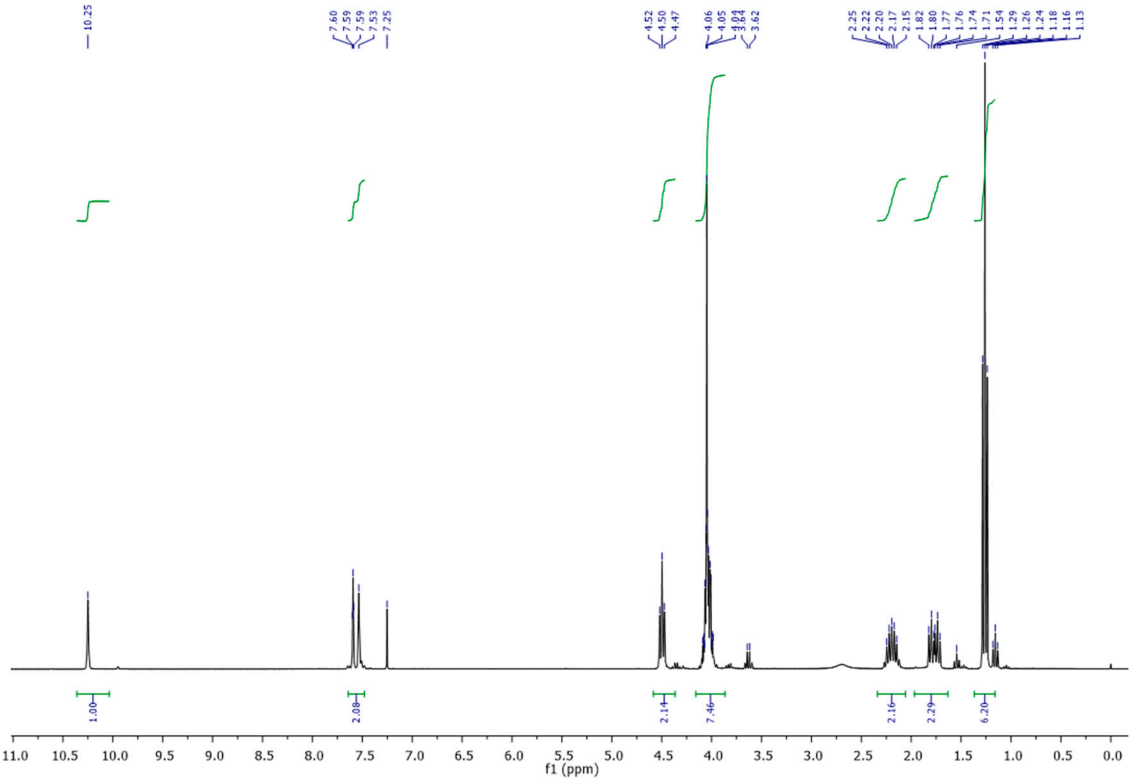

Figure S3. ^{1}H NMR spectra of ImPE.

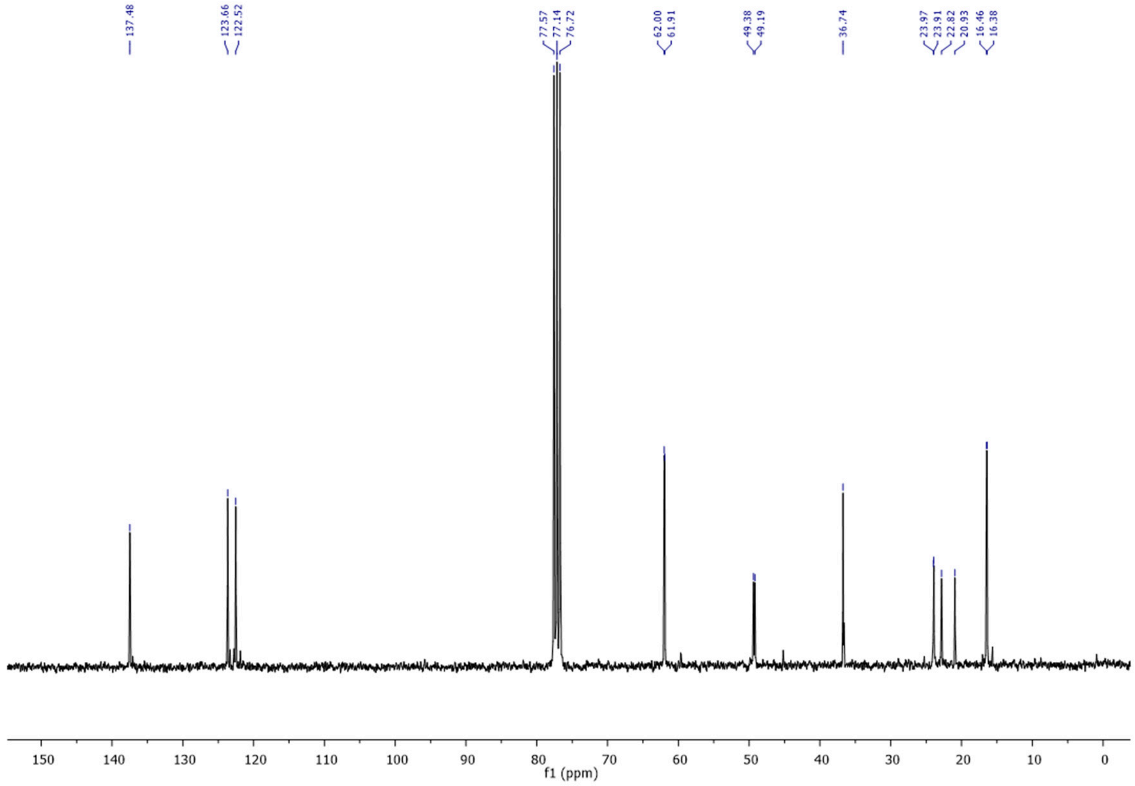

Figure S4. ^{13}C NMR spectra of ImPE.

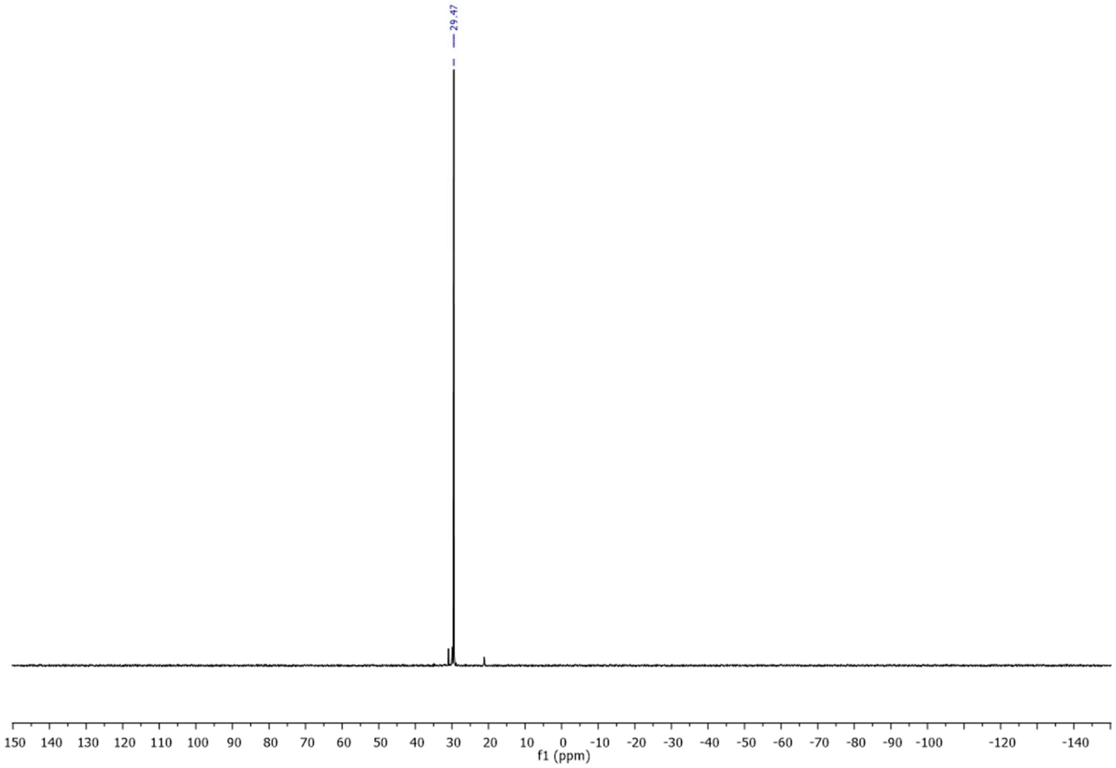

Figure S5. ^{31}P NMR spectra of ImPE.

ImTMSP

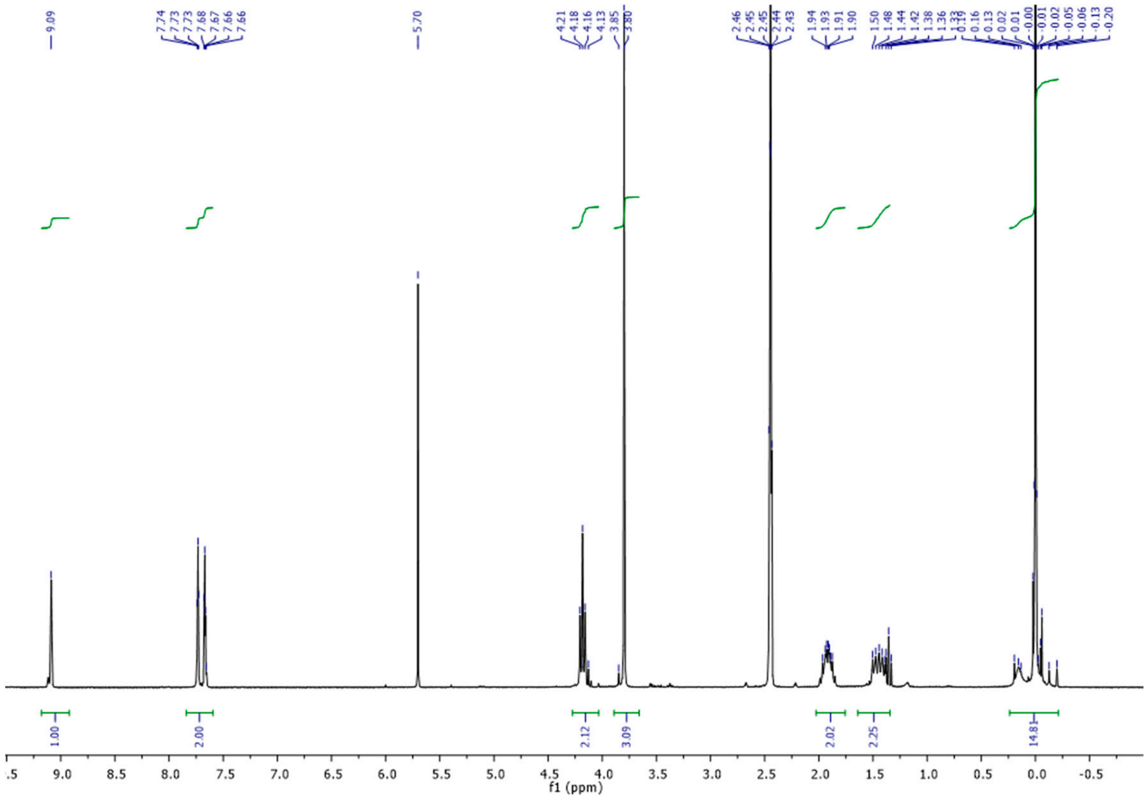

Figure S6. ^{1}H NMR spectra of ImTMSP.

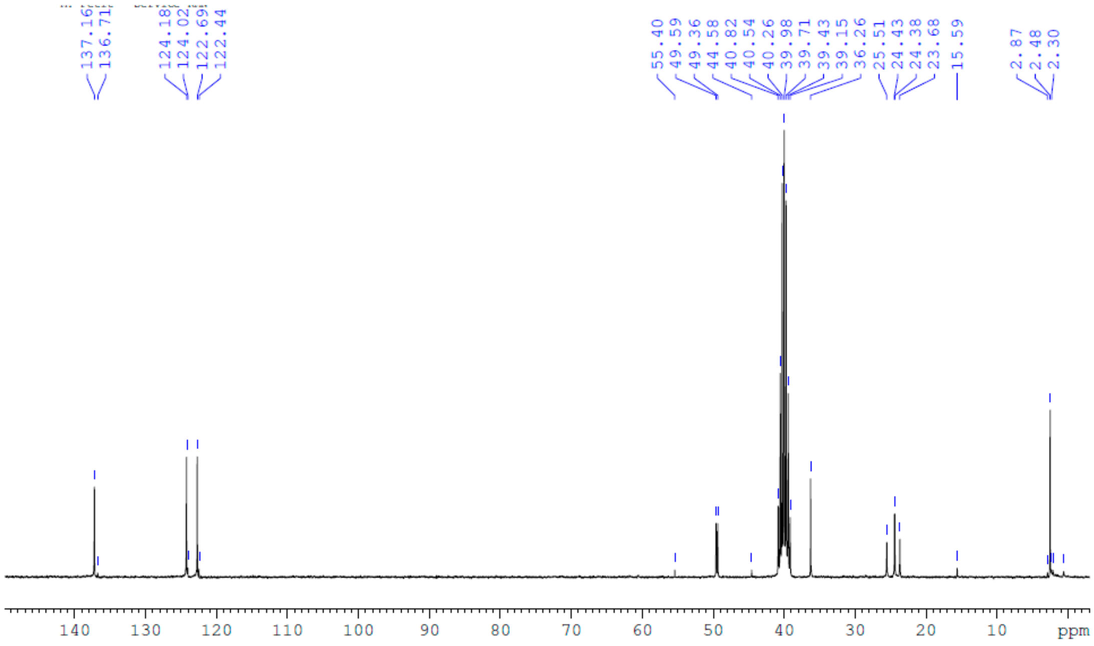

Figure S7. ^{13}C NMR spectra of ImTMSP.

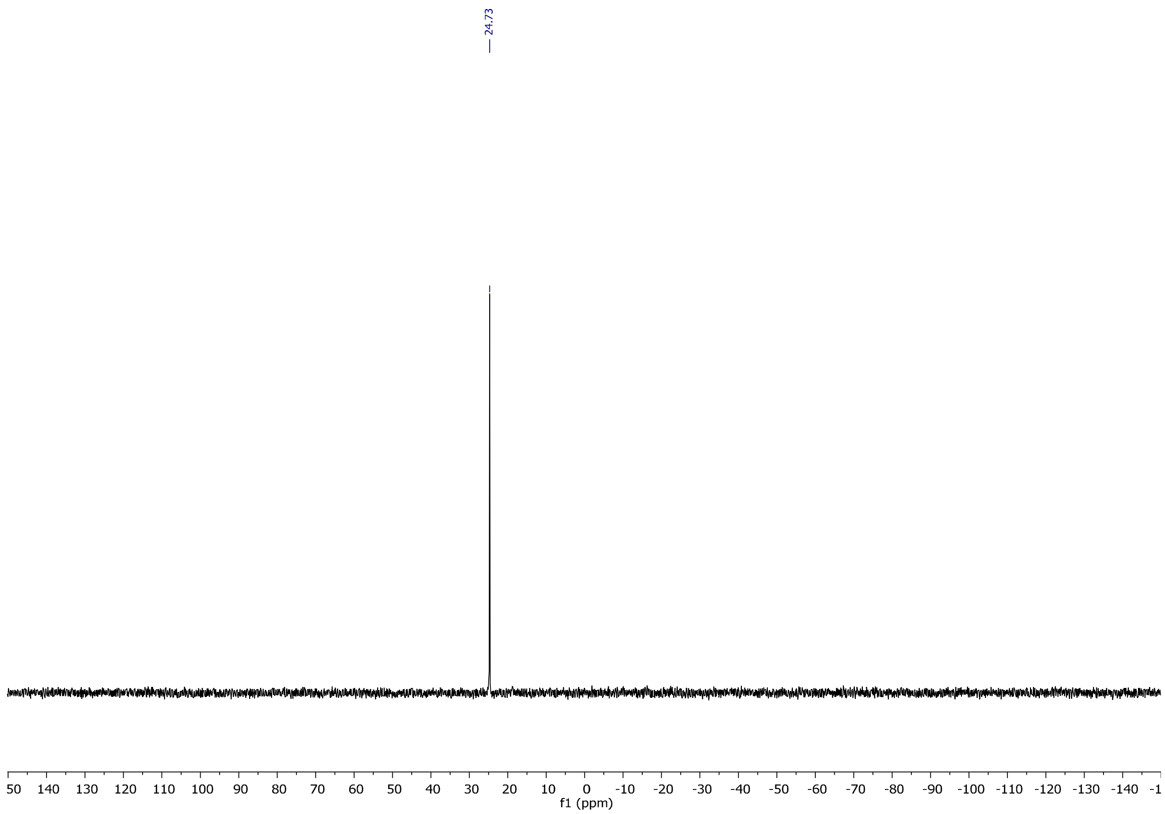

Figure S8. ^{31}P NMR spectra of ImTMSP.

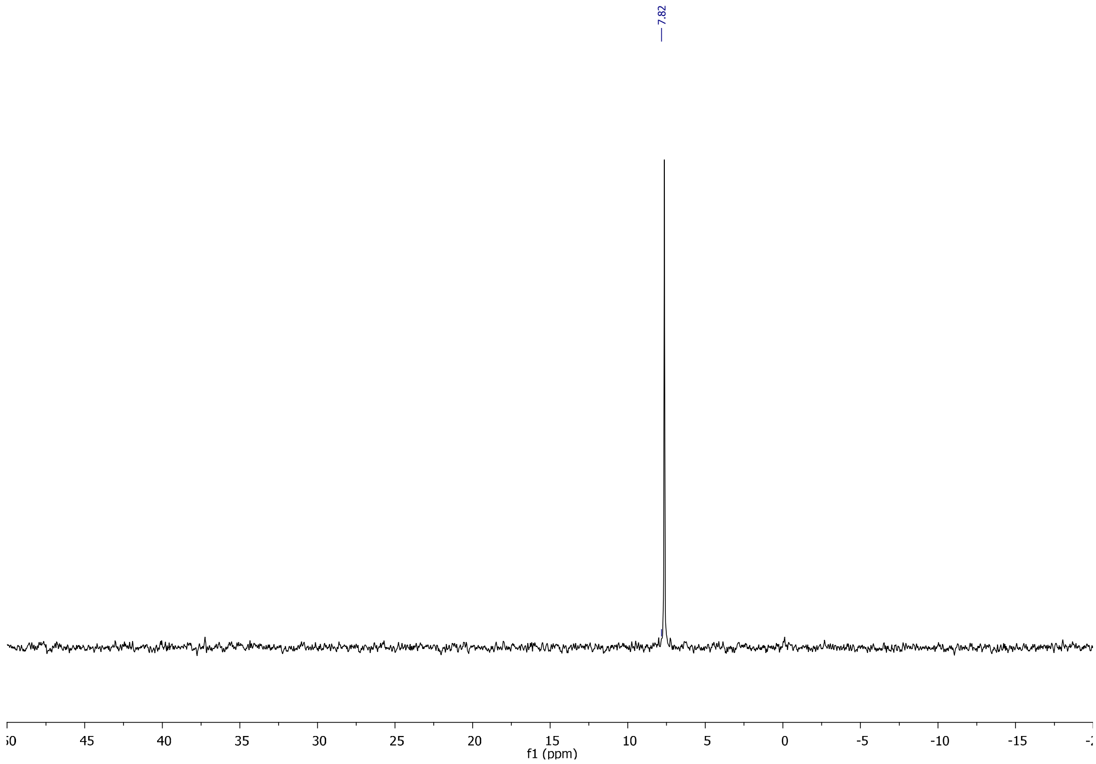

Figure S9. ^{29}Si NMR spectra of ImTMSP.

5. FTIR Spectra of the Ionic Liquids in ATR Mode and DFT Calculated Spectra

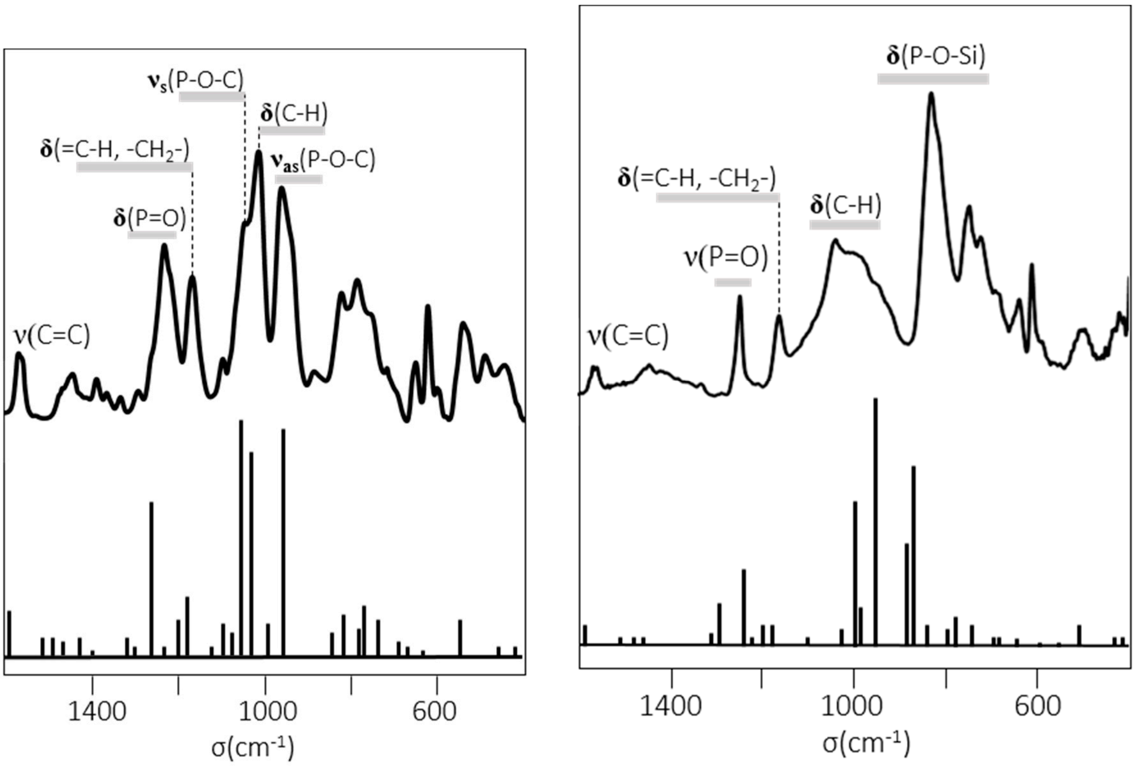

(**a**) (**b**)

Figure S10. Portion of the experimental IR spectra of the ionic liquid (IL): (**a**) ImPE; (**b**) ImTMSP and representation of the DFT-calculated frequencies for these ILs.

Computational Method:

The calculations were performed with the help of Density Functional Theory (DFT) with B3LYP levels, using 6-311G(2d,p) as basis set on Gaussian 09 program package.Geometry optimization and harmonic vibrational frequencies were calculated at the same level DFT. The predicted wavenumbers correspond to the isolated molecular state (the experimental wavenumbers correspond to a liquid state spectrum). No scaling factors were applied. A detailed interpretation of the vibrational spectra of these compounds have been made.

6. Physisorbed Sample

Physisorption Conditions. The "physisorption" conditions of modification is summarized in Table 1. Grafting solutions of ImPE was prepared in CH_2Cl_2 by dissolving 0.6 mmol of the phosphorus ionic liquid, which corresponds to a 1-fold excess. 5 mL of the grafting solution was placed in a glass bottle. 400 mg of γ-alumina powder stored under argon was added and the bottle was closed with a Teflon caps. The suspension was maintained at 30 °C during 1h30. Then, the supernatant was removed and the resulting powder was dried under vacuum (5–10 mbar) at 70°C for ~16 h. The sample was called **ImPE-Ph** (wt % P: 1.6 ± 0.04)

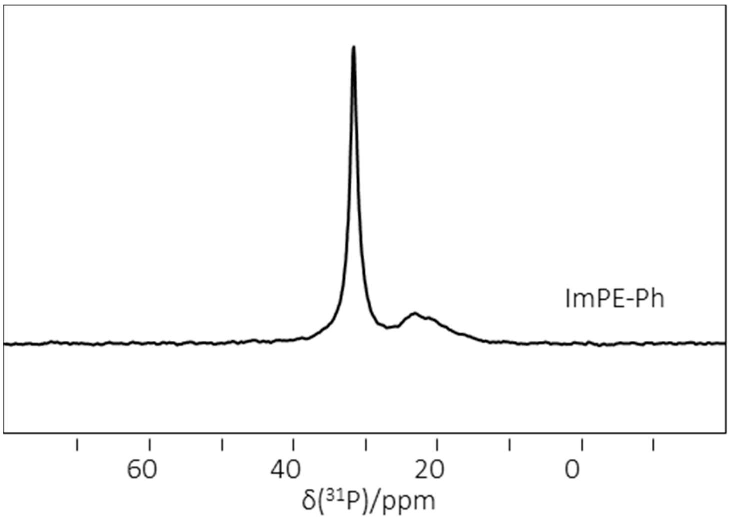

Figure S11. ^{31}P solid-state NMR spectra of ImPE-Ph.

7. XRD of γ-Alumina and Grafted Powders

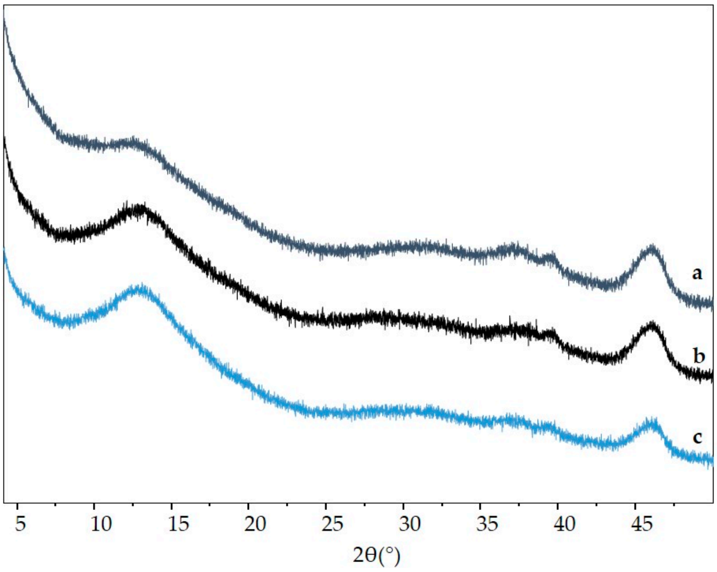

Figure S12. Examples of powder XRD diffractograms: (a) starting γ-alumina powder; and grafted sample realized in: (b) forced condition (ImPE4); (c) standard condition (ImTMSP4).
